# Solar keratoma: An atypical case

**DOI:** 10.4102/jsava.v86i1.1257

**Published:** 2015-09-18

**Authors:** Sean M. Miller, Ralph H. Katzwinkel

**Affiliations:** 1 Summerveld Equine Hospital, Gillitts, South Africa

## Abstract

This case report shows that keratomas can occur in both hind feet of equine patients and should be considered as a diagnosis for long-standing, intermittent lameness localised to the hooves. A Thoroughbred racehorse presented with long-standing abscessation of the right hind hoof. Owing to the long-standing nature, the abscess draining tract was surgically explored. A focal mass was found within the solar horn. Histopathology revealed the mass to be a keratoma. A similar mass was removed from the left hind hoof a few months later after similar presenting signs. This case shows that keratomas can occur in more than one hoof within a short period and should be considered a differential diagnosis for long-standing lameness localised to the hoof.

## Introduction

This case report describes that keratomas can occur in both hind feet of equine patients and should be considered as a diagnosis for long-standing, intermittent lameness localised to the hooves.

Most authors consider keratomas to be slow-growing, benign, hyperplastic masses that occupy space in the affected anatomic area (Hamir, Kunz & Evans 1992; Honnas, Dabareiner & McCauley 2003; O’Grady & Horne 2001) and are associated with variable clinical signs (Redding & O’Grady 2012). Although uncommon as a cause of lameness, keratomas are clinically well documented (Floyd & Mansmann 2007; Hamir *et al.* 1992; Lloyd *et al.* 1988; Moyer 2008). The most common presenting complaint is recurrent lameness and abscessation of the hoof (Pickersgill 2000). Lameness results from pressure on the dermal laminae (Stashak & Hill 1996) and may worsen after shoeing (Schulze, Weinberger & Maraki 2006), potentially leading to a draining tract developing in the white line (Chaffin, Carter & Sustaire 1989; Honnas *et al.* 2003; Lloyd *et al.* 1988; Wagner, Balch-Burnett & Merritt 1986).

Determining the exact cause of keratomas often proves problematic (Chan & Munroe 1997; Honnas *et al.* 2003), but previous trauma or chronic irritation may play a role (Christman 2008; Floyd & Mansmann 2007; Furst & Lischer 2006; Lloyd *et al.* 1988; Wagner *et al.* 1986). For example, lateromedial hoof imbalance and unusual shoeing place abnormal stress on the hoof, which may lead to chronic irritation and subsequent keratoma formation (Back, Schie & Bosch 2007; McDiarmid 2007). Keratomas are anecdotally reported to occur less commonly in arid regions (Cullimore & Booth 2010). There appears to be no age or sex predilection (Hamir *et al.* 1992; Lloyd *et al.* 1988; Mair & Linnenkohl 2012).

In a normal hoof, the keratinised epidermal laminae interlock with the dermis originating from the coronary band (Bowker 2003; Hamir *et al.* 1992) and, once formed, migrate downwards almost unchanged (Bowker 2003). The sole also contains keratinised horn (Bowker 2003; O’Grady & Horne 2001). Hoof horn normally reacts to loading stress by producing an increased amount of keratinised horn as a protective measure (Back *et al.* 2007) and adapts accordingly by changing its structure when formed (Bowker 2003; Stashak & Hill 1996).

Two typical forms of keratomas have been described, namely a cylindrical and a more discrete, spherical form (Cullimore & Booth 2010; McDiarmid 2007). In the front hoof, the cylindrical form usually occurs as a solitary lesion at the toe or quarter (Boys Smith *et al.* 2006; Mair & Linnenkohl 2012; McDiarmid 2007), whereas multiple locations have been reported in a hind hoof (Christman 2008).

Radiographically, a keratoma presents as a distinct semilunar notch with a smooth sclerotic rim on the mid or distal dorsal phalanx 3 (P3), which distinguishes it from an infectious process (Boys Smith *et al.* 2006; Butler *et al.* 2000; Farrow 2006; Honnas 1997). Several authors have suggested that surgical exploration and histopathology are the only methods available for accurate diagnosis (Hamir *et al*. 1992; Lloyd *et al.* 1988; Moyer 2008; Pickersgill 2000), as radiography may be inconclusive because of the soft tissue detail limitations (Boys Smith *et al.* 2006; Honnas 1997; Schulze *et al.* 2006), the location of the keratoma or the extent of growth (Chan & Munroe 1997). Differential diagnoses include squamous cell carcinoma, malignant melanoma, mast cell tumour, glomus tumour and fibroma (Brounts *et al.* 2008; Durham & Walmsley 1997; Moyer 2008; Valentine *et al.* 2000), as well as septic and nonseptic pedal osteitis (Cauvin & Munroe 1998; Chaffin *et al.* 1989; Chan & Munroe 1997). Recently, however, magnetic resonance imaging (MRI) has allowed for easier diagnosis of keratomas of atypical appearance (Mair & Linnenkohl 2012).

Keratomas may lead to recurrent submural or subsolar abscesses (Ange 2010; Boys Smith *et al.* 2006) and are often associated with local secondary infection (Boys Smith *et al.* 2006; Butler *et al.* 2000; Lloyd *et al.* 1988). However, it has also been suggested that keratomas rather mimic an abscess (Cullimore & Booth 2010; Wagner *et al.* 1986). Keratomas commonly consist of poor-quality horn that easily decays, which allows bacteria and fungi to enter the hoof (Furst & Lischer 2006), resulting in a necrotic core (Honnas *et al.* 2003).

Surgical excision of all abnormal tissue is currently the preferred treatment (Honnas 1997; Honnas *et al.* 2003; Pickersgill 2000; Redding & O’Grady 2012), either by dissection or by curettage (Moyer 2008), followed by therapeutic shoeing (Ange 2010). Prognosis after uncomplicated complete surgical excision is good (Boys Smith *et al.* 2006; Dyson 2003; Floyd & Mansmann 2007; McDiarmid 2007; Wagner *et al.* 1986) and is associated with significantly better outcome than conservative treatment (83% vs 43% success rate) (Furst & Lischer 2006).

## Management and outcome

### History

A four-year-old Thoroughbred gelding racehorse presented with mild, intermittent lameness (grade 1, as defined by the American Association of Equine Practitioners scale [Anon 1991]) in the right hind limb. Hoof testers placed on the lateral quarter of the hoof induced discomfort and evoked a reaction. The initial diagnosis was that of a subsolar abscess or sole bruising. The hoof was treated topically with a poultice of acriflavine, glycerine and magnesium sulphate and systemic anti-inflammatories (phenylbutazone: 2.2 mg/kg body weight) were administered per os twice a day (Virbac RSA, Halfway House). The solar abscess and associated lameness recurred repeatedly over a four-month period. As part of the diagnostic work-up, a regional (lateral sesamoid) nerve block of the affected limb was performed, resulting in resolution of lameness. The horse had normal hoof conformation and was shod with 1° heel wedges on its hind hooves.

### Physical examination

When examined, the horse was shod with steel shoes with 1° heel wedges. The hoof was considered within normal limits with regard to size, shape and angle to the ground and pastern. The structure of the hoof appeared normal, with no deviations of the white line. The persistent cycle of assumed abscessation and resolution in the lateral quarter, and the positive reaction to hoof testers in the same location, suggested a sequestrum of unknown origin or possible foreign body. At the time of examination, there was a draining tract in the lateral quarter. Surgical exploration of the tract was performed. Radiographs were not taken initially.

### Procedure

**FIGURE 1:**
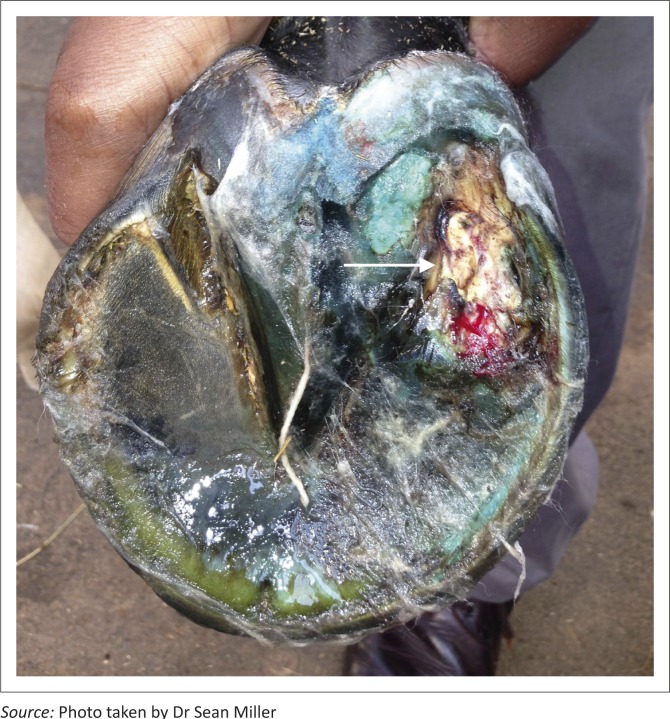
Right hind hoof of a four-year-old Thoroughbred gelding racehorse, showing the location of a keratoma in the lateral quarter. This photo was taken 5 days post-removal.

The procedure was performed with the horse in a standing position. Sedation was achieved using a combination of detomidine (0.01 mg/kg body weight; Domosedan, Pfizer, South Africa) and butorphanol (0.05 mg/kg body weight; Torbugesic, Fort Dodge Animal Health, Fort Dodge) administered intravenously. The hoof was desensitised with an abaxial plantar nerve block at the level of the sesamoid bones. Standard and looped hoof knives were used to pare away solar horn along the draining tract in the lateral quarter of the right hind hoof. As the tract enlarged, an irregular spherical mass was observed within the cavity. The mass was loosely attached to the surrounding tissue and was easily removed. The wound cavity was curetted to remove all necrotic tissue, packed with gauze swabs soaked in an astringent (copper sulphate) and then bandaged with a water-resistant bandage. The mass was preserved in 10% buffered formalin and submitted for histopathological examination. Figure 1, taken 5 days after the procedure, shows the location of the keratoma.

### Aftercare

The horse received a course of phenylbutazone (Virbac RSA, Halfway House) for 5 days post procedure (2.2 mg/kg body weight per os once a day). The bandage was replaced every 3 days for 2 weeks and then weekly for a further 2 weeks, repeating the initial packing with copper-sulphate-soaked gauze each time. Granulation tissue was evident after the second bandage change and the tissue keratinised, becoming horn like within a few weeks. At 4 weeks post-removal, a bar shoe was fitted and a light dressing was maintained on the cavity and changed every second day. Systemic antimicrobials were not administered. Owing to the histopathological findings, lateral, dorsopalmar, dorsoproximal–palmarodistal oblique and palmaroproximal–palmarodistal oblique radiographic views were taken of the hoof to determine whether P3 was affected and confirm resolution. No radiographic abnormalities were detected on P3.

### Histopathology

**FIGURE 2:**
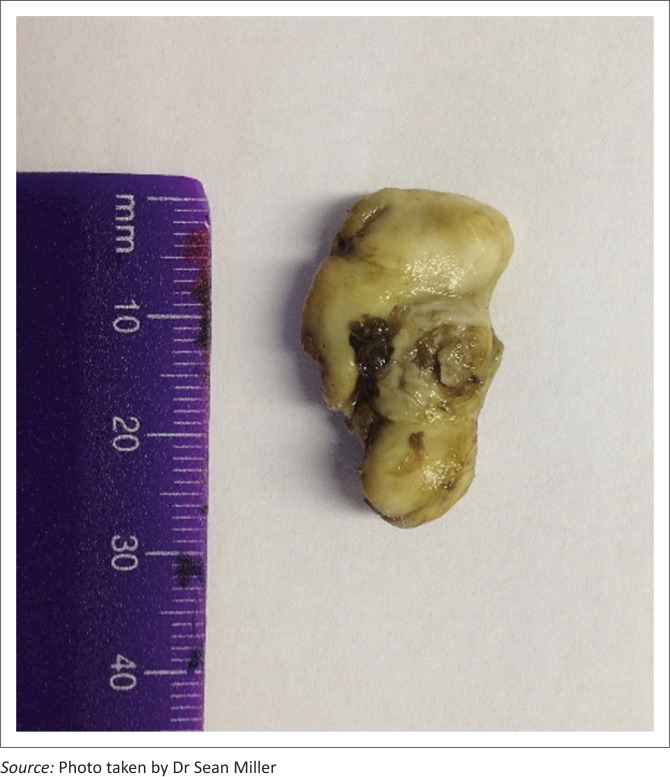
The keratoma that was removed from the right hind hoof of a four-year-old Thoroughbred gelding racehorse.

The irregular spherical mass of 3 cm × 2 cm × 1 cm (Figure 2) was submitted for histopathology in 10% buffered formalin. The mass consisted of concentric laminated keratin embedded in solar keratin. The final diagnosis confirmed suspicions of a solar keratoma.

## Discussion

The horse returned to ridden exercise 1 month after the procedure was performed. It then developed a similar subsolar abscess in the medial quarter of the left hind hoof. The abscess recurred repeatedly over a period of 2 months, with associated intermittent lameness (grade 1, [Anon 1991]). Based on the similar nature of the clinical signs, the abscess was investigated surgically. A mass of similar appearance (2 cm × 1 cm × 1 cm) was found in the hoof. It was more firmly attached to the underlying hoof tissue than the one in the right hind hoof. The mass was removed and submitted for histopathology. However, histopathological findings were inconclusive, possibly owing to the mass having been at an early stage of formation, before maturation could occur. The mass was suspected to be a keratoma.

The horse returned to ridden exercise 1 month after removal of the mass. Five months after returning to ridden exercise, the horse placed second in its second racing start and subsequently won its third start.

This case is unusual because a keratoma was diagnosed in one hind foot and highly suspected in the other hind foot in a fairly short time period. Keratomas are not commonly reported in more than one hoof (Mair & Linnenkohl 2012). Although spherical keratomas have been described in the frog (McDiarmid 2007), sole (O’Grady & Horne 2001) and above the coronary band (Valentine *et al.* 2000) in horses, they are uncommon in these locations and seldom reported in the literature (O’Grady & Horne 2001).

There was no evidence of previous trauma to either hoof. As shown in this case, radiographs were not useful as a diagnostic tool, possibly owing to the specific location of the mass. As the solar horn is not as rigid as the dorsal wall, the growing keratoma likely resulted in less pressure necrosis on P3 (Gasiorowski & Richardson 2011). Modalities such as nuclear scintigraphy or MRI may have been more helpful (Floyd & Mansmann 2007), as focal thinning of the sole may be seen (O’Grady & Horne 2001) and may be more readily visible on MRI than on radiographs (Mair & Linnenkohl 2012). MRI would also allow earlier detection, prompt treatment and good surgical planning (Barrett & Subrod 2008; Dyson 2003; Mair & Linnenkohl 2012), although the costs are prohibitive (Schulze *et al.* 2006). Ultrasound would have been a possible diagnostic aid if the lesion had been above the coronary band (Floyd & Mansmann 2007; Honnas *et al.* 2003).

Solar keratomas are difficult to diagnose and should be considered when long-standing abscessation or chronic low-grade lameness attributed to the hoof are the presenting complaints. As shown in this case, it is possible that keratomas can occur in both hind hooves of a horse. This suggests that keratomas can occur on any hoof and can potentially occur in multiple hooves of a single horse.
